# Molecular Characterization of Larval Peripheral Thermosensory Responses of the Malaria Vector Mosquito *Anopheles gambiae*

**DOI:** 10.1371/journal.pone.0072595

**Published:** 2013-08-07

**Authors:** Chao Liu, Laurence J. Zwiebel

**Affiliations:** 1 Department of Biological Sciences, Vanderbilt University, Nashville, Tennessee, United States of America; 2 Program in Developmental Biology, Department of Pharmacology, Vanderbilt Brain Institute and Institutes of Chemical Biology and Global Health, Vanderbilt University Medical Center, Nashville, Tennessee, United States of America; United States Department of Agriculture, Beltsville Agricultural Research Center, United States of America

## Abstract

Thermosensation provides vital inputs for the malaria vector mosquito, *Anopheles gambiae* which utilizes heat-sensitivity within a broad spectrum of behaviors, most notably, the localization of human hosts for blood feeding. In this study, we examine thermosensory behaviors in larval-stage *An. gambiae*, which as a result of their obligate aquatic habitats and importance for vectorial capacity, represents an opportunistic target for vector control as part of the global campaign to eliminate malaria. As is the case for adults, immature mosquitoes respond differentially to a diverse array of external heat stimuli. In addition, larvae exhibit a striking phenotypic plasticity in thermal-driven behaviors that are established by temperature at which embryonic development occurs. Within this spectrum, RNAi-directed gene-silencing studies provide evidence for the essential role of the Transient Receptor Potential sub-family A1 (TRPA1) channel in mediating larval thermal-induced locomotion and thermal preference within a discrete upper range of ambient temperatures.

## Introduction

*Anopheles gambiae sensu stricto* (Diptera: Culicidae) is the principal sub-Saharan vector of human malaria that causes over a million deaths annually [1]. As is true for all mosquitoes, *An. gambiae* goes through pre-adult development spanning egg, larval and pupal life stages in aqueous environments. This period typically lasts between 5 and 14 days, depending on population density, food level and water temperatures in larval habitats [2]. Although frequently overlooked, it has long been appreciated that a significant degree of vector control is accomplished through regulation of larval populations. Indeed, efficient regional eradication of malaria has been achieved primarily through larvicidal intervention [3]. In addition, due to their aquatic lifestyle and considerably less complex nervous system, immature *An. gambiae* represents a more tractable stage for the basic study of various physiological and sensory processes [4]. Indeed, previous studies have taken advantage of both simplicity and reproducibility of larval *An. gambiae* to explore the basic principles underlying adult olfactory-driven responses, which also serve as a foundation for further exploration of other aspects of larval sensory biology [5,6].

Mosquitoes are poikilotherms and as a result, are incapable of maintaining thermal homeostasis [7]. Consequently, aquatic larvae rely on their ability to sense and respond to temperature cues for several survival-dependent behaviors in response to local temperature fluctuations. These include the ability to navigate through rapidly changing water temperatures in larval habitats that are alternately exposed to sunlight and darkness during day/night cycles [8]. Therefore, the functional characterization of thermal sensitivity in mosquito larvae would provide insights into these processes as well as potentially inform our understanding of the adult sensory system and facilitate the development of novel approaches that are designed to modulate larval thermosensory behaviors to elicit larvicidal activity.

While the molecular mechanisms underlying thermosensation in *An. gambiae* larvae remain largely unexplored, earlier studies have established the role of *An. gambiae* TRPA1 (hereafter, AgTRPA1), a member of the Transient Receptor Potential family of sensory proteins, in conferring sensitivity of adult peripheral thermosensory pathways to increasing temperatures from 25 to 37°C [9]. This is consistent with studies in other insects suggesting that TRPA1 represents an evolutionarily ancient multimodal channel protein that is responsible for sensing temperatures across the warm and/or hot range [10–12]. In order to continue the exploration of peripheral thermosensation and in particular, the role of AgTRPA1 in this context, we now focus on late-stage larvae that represents a critical developmental window in establishing vectorial capacity of *An. gambiae*. These studies have characterized the causal relationships between ambient temperature and larval behavior and more importantly, identify AgTRPA1 as a narrowly tuned peripheral high temperature sensor in larvae that is crucial for regulating mobility as well as thermal preference.

## Results

### Kinetic larval response to ambient temperatures

In order to understand the molecular processes by which mosquito larvae sense external thermal signals, we first investigated the impact of ambient temperature on larval locomotion. To accomplish this we assayed overall larval mobility as a mechanism to assess larval responses to a range of increasing water temperatures. We obtained uniform heating conditions by programming two Peltier devices to the same temperature set point (See methods). In this manner we were able to precisely control the water temperature within a glass petri dish that was placed upon the aluminum sheet, as monitored by a digital heat probe (HCC-100A DAGAN Corporation). Individual *An. gambiae* 4^th^ instar larvae (reared at 27° C, see methods) were then introduced at the center point of the arena and allowed to swim at will for 5mins subsequent to a 15s acclimation period.

**Figure 1:**
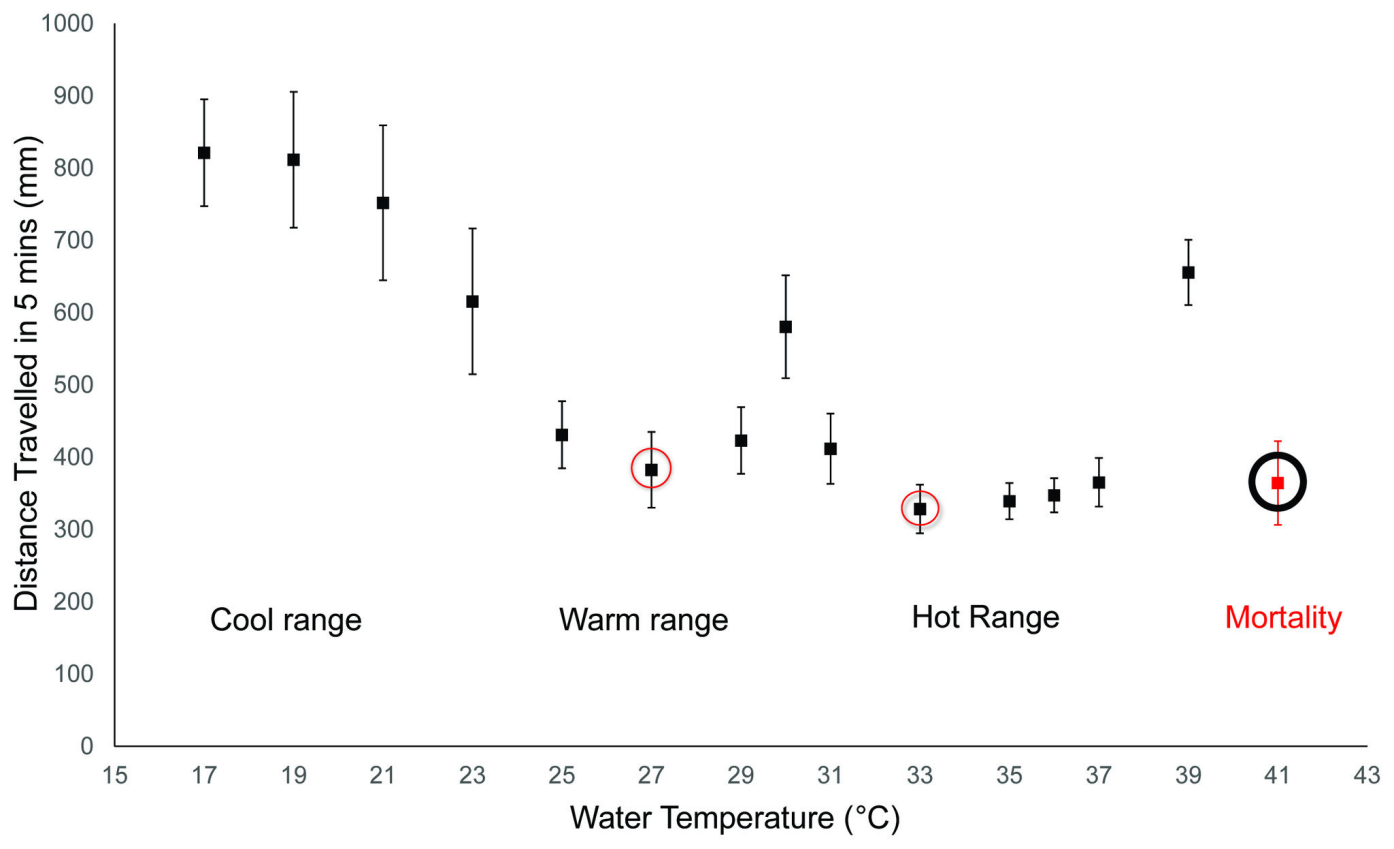
Thermal-induced mobility in WT 4^th^ instar *An*. ***gambiae* larvae**. Arithmetic means ± standard error of the mean (S.E.M) of total distance travelled by individual larva in 300s were plotted (n≥15). Red circle indicates the two individual temperatures that generated lowest larval mobility in the neighboring temperature ranges (27 and 33°C) while black circle shows the temperature at which larvae experienced morbidity/death after 2-3 mins of assaying (41° C), thus the total distance was calculated based on the time frame before larval mortality.

In these assays (Figure 1), *An. gambiae* larvae exhibited relatively high levels of mobility (total distance > 750mm) in cold temperatures (17-21°C); the level of overall movement gradually decreased as ambient temperatures approach 27° C (total distance: 382.7mm). Further increasing water temperature resulted in larval mobility returning to a moderate level at approximately 30° C (total distance: 580.5mm), and then decreasing again as conditions enter the hot temperature range (33-37°C) (total distance<350mm). Not surprisingly, once the water temperature reached 39° C, larval locomotion increased significantly (total distance: 655.7mm) although conditions by 41° C no longer supported viability while morbidity and/or mortality was evident after 2-3mins of assaying. These experiments indicate that *An. gambiae* larvae are capable of recognizing and responding to varying ambient temperatures, leading to distinctive kinetic responses.

### Thermal-induced kinesis reveals larval thermal preferences

The kinetic responses of *An. gambiae* larvae to individual temperatures are consistent with pre-described patterns of attractive or repulsive stimuli [13]. When challenged with a non-directional stimulus such as ambient temperature, faster movements of the subject, or positive orthokinesis, may imply behavioral aversion to the stimulus while slower rates of movement (negative orthokinesis) is consistent with attractive cues [13,14].

**Figure 2:**
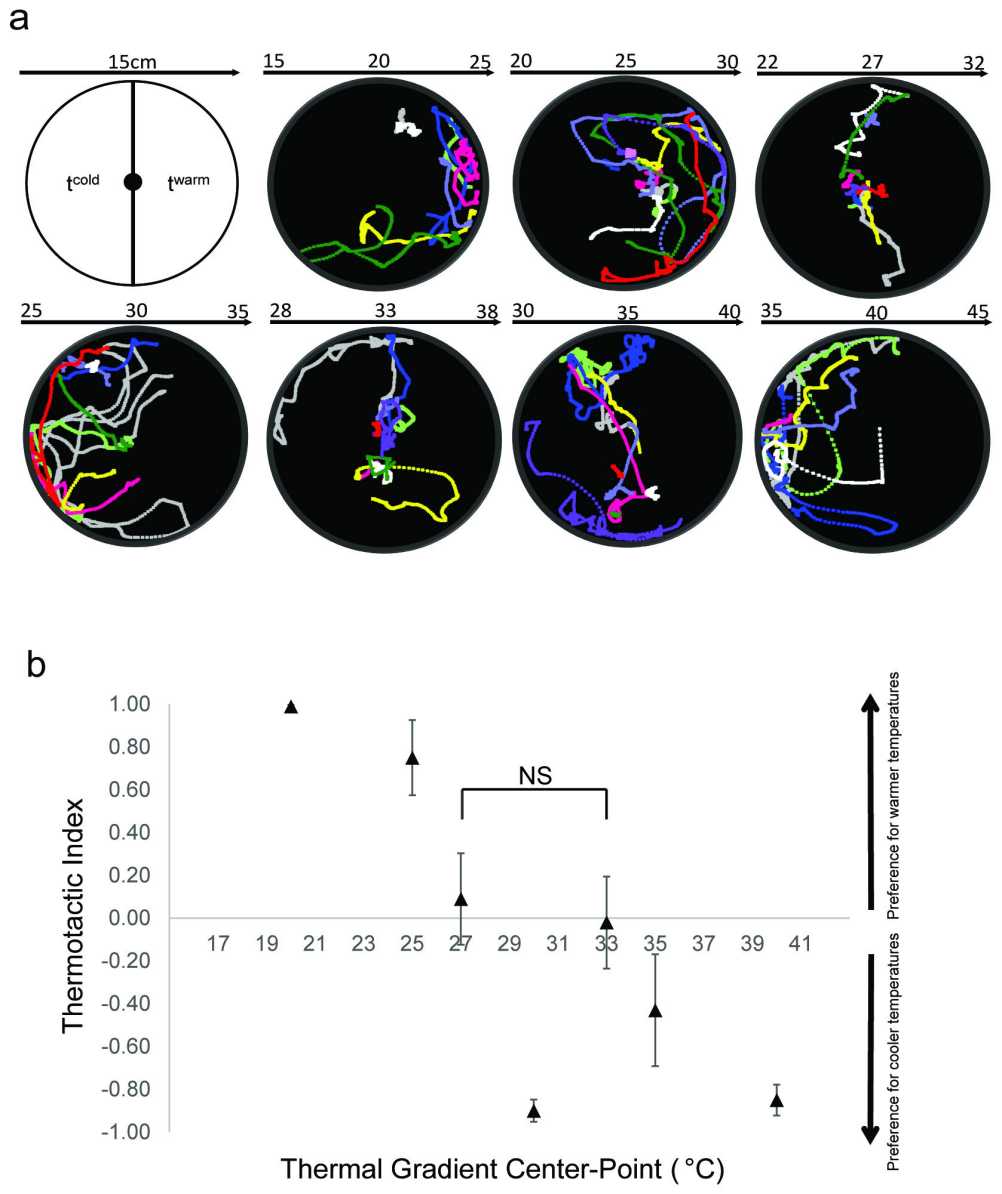
Thermal preferences of WT 4^th^ instar *An*.*gambiae* **larvae**. **a**) Individual *An. gambiae* larva was introduced into the center of the behavioral arena and recording started following a 15s acclimation period. Swimming trajectories from a minimum of 10 individual larvae reared at 27° C were superimposed. Each color represents a separate trial. **b**) Arithmetic means ± S.E.M (n≥10) of thermotactic indices in 7 different thermal gradients were plotted. Mann–Whitney *U* test was used to compare thermotactic indices at 27 and 33°C with a *p* value > 0.05.

In this light, it is noteworthy that the recorded larval mobility achieved the lowest values at 27° C and 33° C when compared to movement rates at neighboring temperature ranges (17 to 30°C and 30 to 39°C, respectively). This phenomenon raises the hypothesis that *An. gambiae* larvae in this study display a preference for ambient temperatures around 27 and 33°C. To verify this we explored their inherent thermal preferences on a linear temperature gradient (0.67°C/cm). A total of seven gradients were selected for assessment so as to encompass a range of cold (20° C), warm (25, 27, 30°C), hot (33, 35° C) and ultra-hot (40° C) center-point temperatures (Figure 2a). Of these, both thermal gradients across 22-32°C (center point 27° C) and 28-38°C (center point 33° C) failed to induce apparent thermotactic movements in larvae, which spent virtually the same amount of time in both warm and cool sectors of the arena (TI= -0.03±0.17 and -0.13±0.24, respectively; Figure 2b). In contrast, larvae displayed positive thermotaxis in gradients with center points at 20 and 25°C (TI=0.95±0.04, 0.62±0.18, respectively) and negative thermotaxis in gradients of 30 and 40°C center point (TI=-0.9±0.04, -0.91±0.08, respectively; Figure 2b). Lastly, weak negative thermotaxis was observed in larvae exposed to thermal gradient with center point at 35° C (TI= -0.35±0.22; Figure 2b).

These data correlate with the larval kinesis at discrete ambient temperatures and suggest *An. gambiae* larvae are capable of distinguishing small variances presented across a linear temperature gradient and moreover, they execute directional movements towards preferred temperatures. Surprisingly, *An. gambiae* larvae display thermal preferences to two distinct temperatures that are 6° C apart (27 and 33°C). It is also notable that cooler half of the gradient was preferred over warmer side when both 27 and 33°C were present in the same gradient (Figure 2a, 25-35°C panel).

### Plasticity of thermal-driven behavior elicited by An *gambiae* larvae triggered by the shift of cultivation temperature

**Figure 3:**
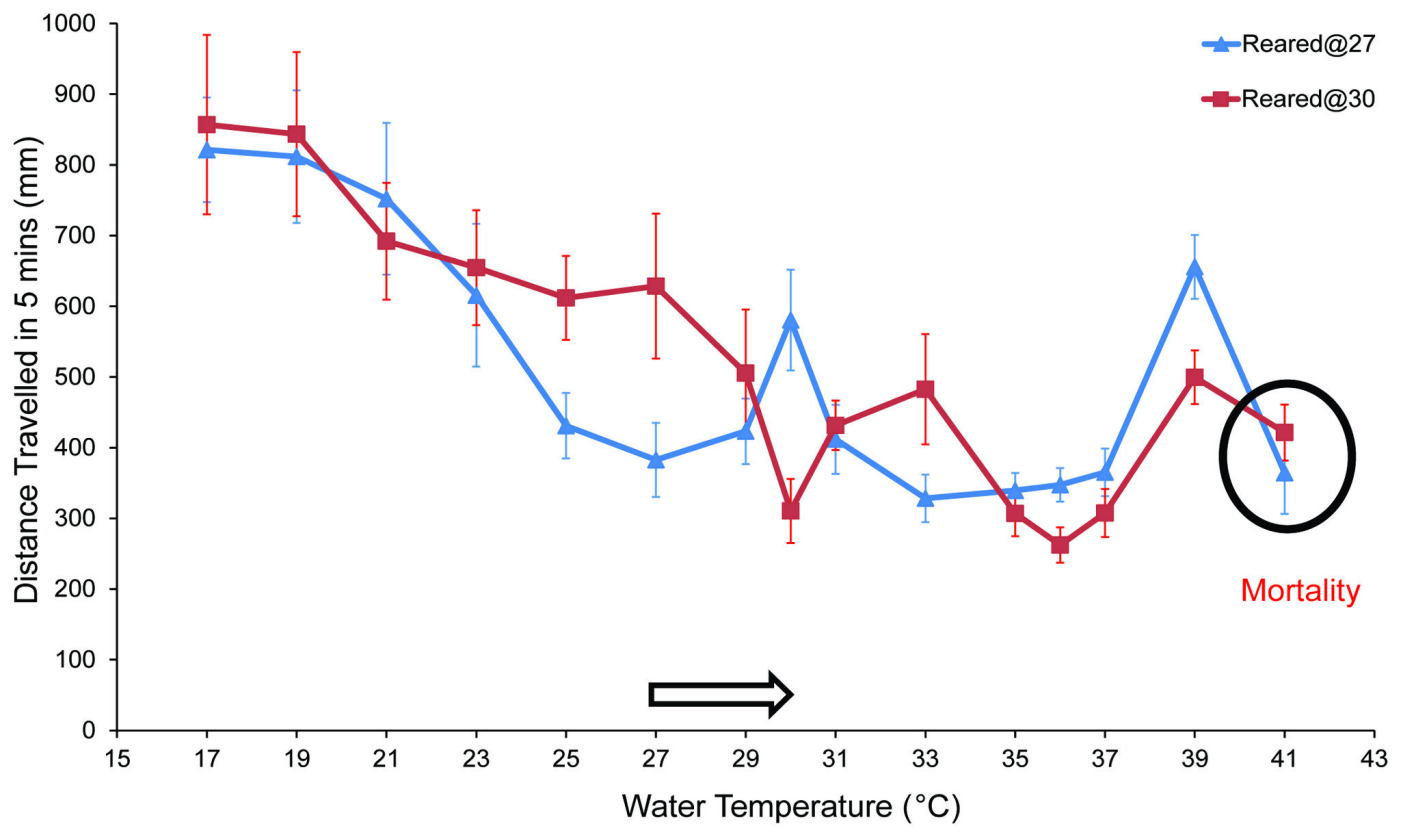
Thermal-induced larval mobility following the shift of cultivation. Arithmetic means ± S.E.M recorded from larvae reared at both 27 and 30°C of total distance travelled in 300s were plotted (n≥12). White arrow shows the shift of cultivation temperature from 27 to 30°C. Black circle shows the temperature at which larval mortality was evident for both 27 and 30°C-reared colony. This figure indicates the change of larval mobility pattern matches the shift of rearing temperature.

**Figure 4:**
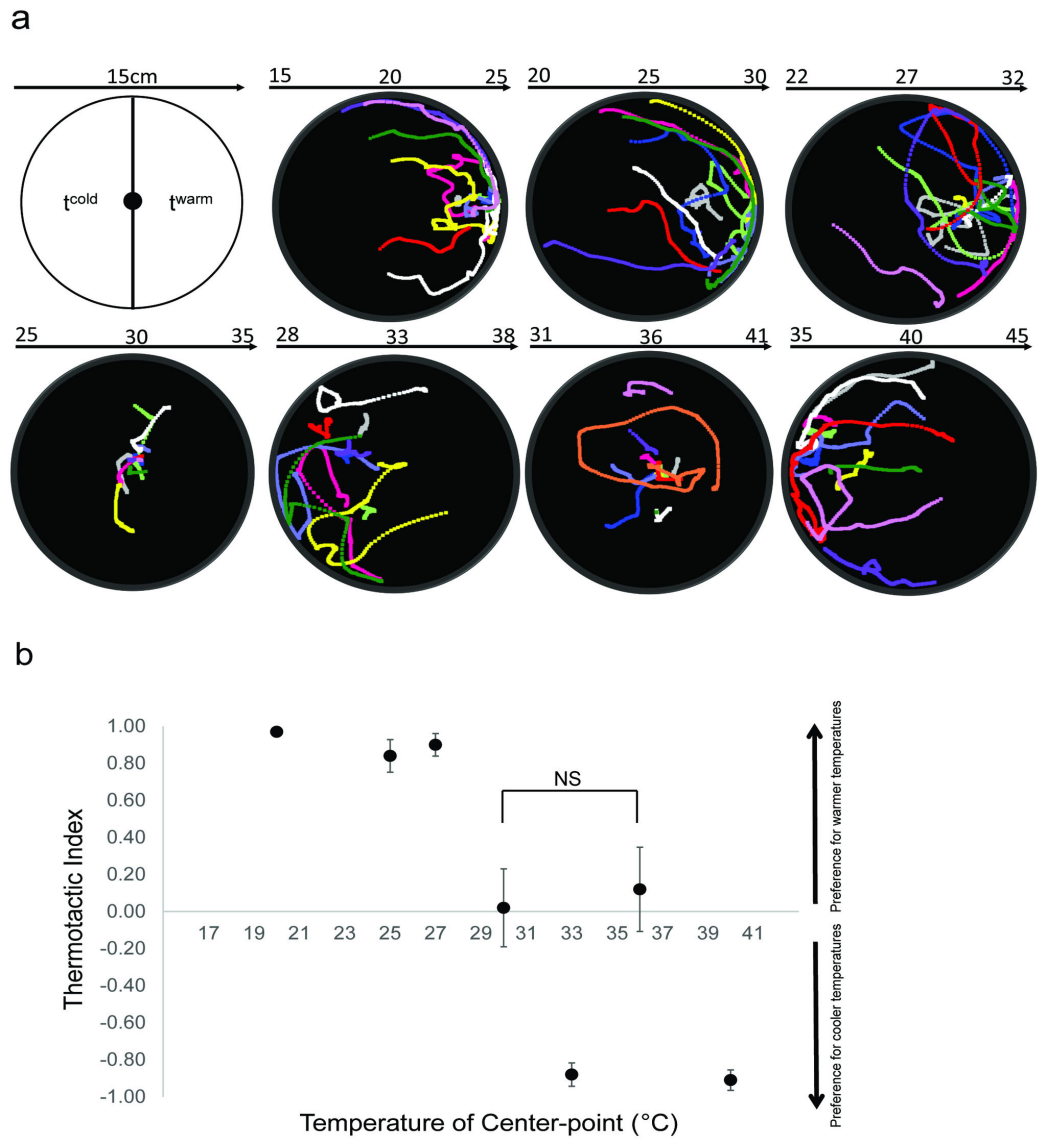
Thermal preferences of WT 4^th^ instar An. ***gambiae* larvae following the shift of cultivation**. **a**) A stack of larval trajectories (n≥10) recorded in 7 different thermal gradients were shown for larvae reared at 30° C. **b**) Larval thermotactic indices ± S.E.M were plotted for larvae reared at 30° C. Mann-Whitney *U* test was used to compare thermotactic indices at 30 and 36°C with a *p* value > 0.05.

The observed behavioral preference towards 27° C by *An. gambiae* larvae raises the question as to whether cultivation temperature plays a role in shaping this aspect of thermal preferences since 27° C indeed, coincides with lab rearing conditions. To examine the effect of cultivation temperature on thermal-driven behavior, we reared larvae at 30° C from eggs obtained from 27° C-colony whilst other rearing conditions (i.e. food, lighting) remained unchanged. Consistent with previous observations, this shift in rearing temperature resulted in no apparent effect other than an increased growth rate such that larvae developed approximately 1 day faster as compared to their counterparts reared at 27° C [15]. However, when L4 larvae reared at 30° C were subject to temperature-kinesis paradigm we observed an approximately 3° C shift in larval mobility responses. Here, mobility gradually decreased towards a 30° C trough (total distance: 310.5mm) and then increased to a moderate level at 33° C (total distance: 482.6mm) before undergoing another reduction between 35°C to 37°C, where 36° C represented the second kinesis trough (total distance: 262.3mm). Mobility once again rose at 39° C (total distance 499.5mm) before the onset of larval mortality at 41° C (Figure 3). Additionally, we detected a 3° C upward shift in larval thermotactic indices relative to larvae reared at 27° C as larvae displayed preference to 30 and 36 instead of 27 and 33°C, respectively (Figure 4). These shifts in behavioral responses precisely matched the 3° C rise in cultivation temperature suggesting that *An. gambiae* larvae define their “thermal space” such that the cold, warm, hot temperature sensors are calibrated based, in part, upon rearing conditions. While a subset of these responses appear to exhibit plasticity, larval behavior within the ultra-hot temperature range (39-41°C) was unaltered by the shift of rearing conditions. This is consistent with the view that aversive responses to noxious temperatures directly associated with lethality would be more rigid.

### AgTRPA1 mediates the larval sensitivity towards hot range temperatures

**Figure 5:**
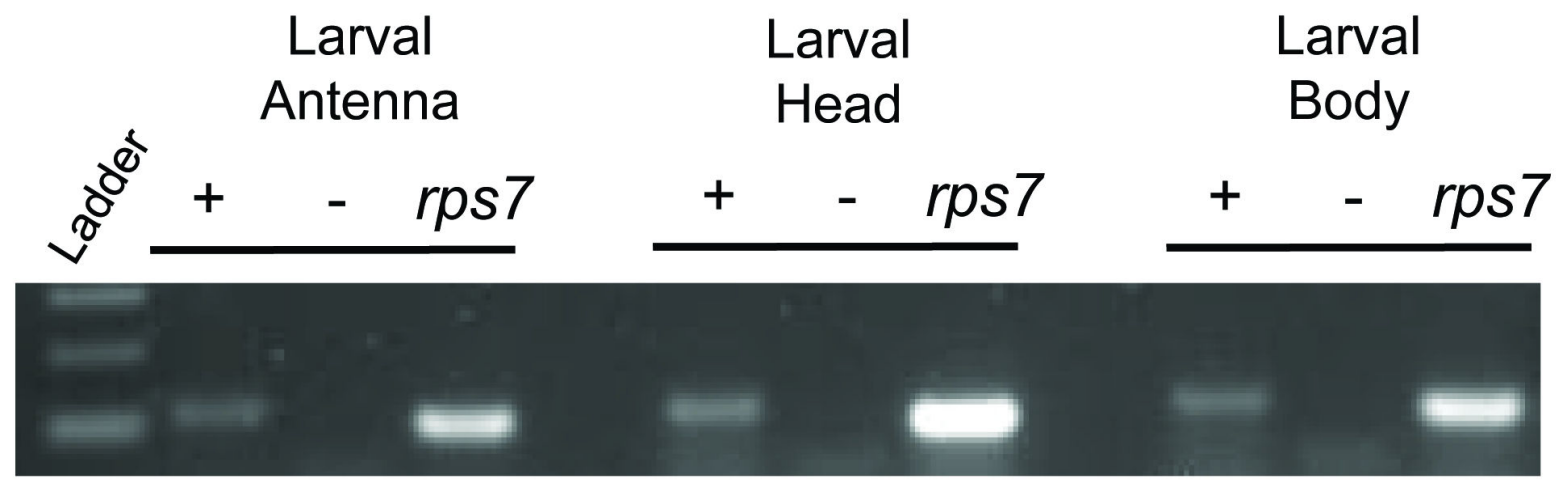
Expression of AgTRPA1 in larval tissues. cDNA libraries from larval antennae, heads and bodies were generated by extracting mRNA followed by *in intro* reverse transcription. *rps7* and *agtrpa1* were amplified using gene-specific primers and run on a 2% agarose gel. “+” or “-“ indicates the presence or absence of reverse transcriptase, respectively.

**Figure 6:**
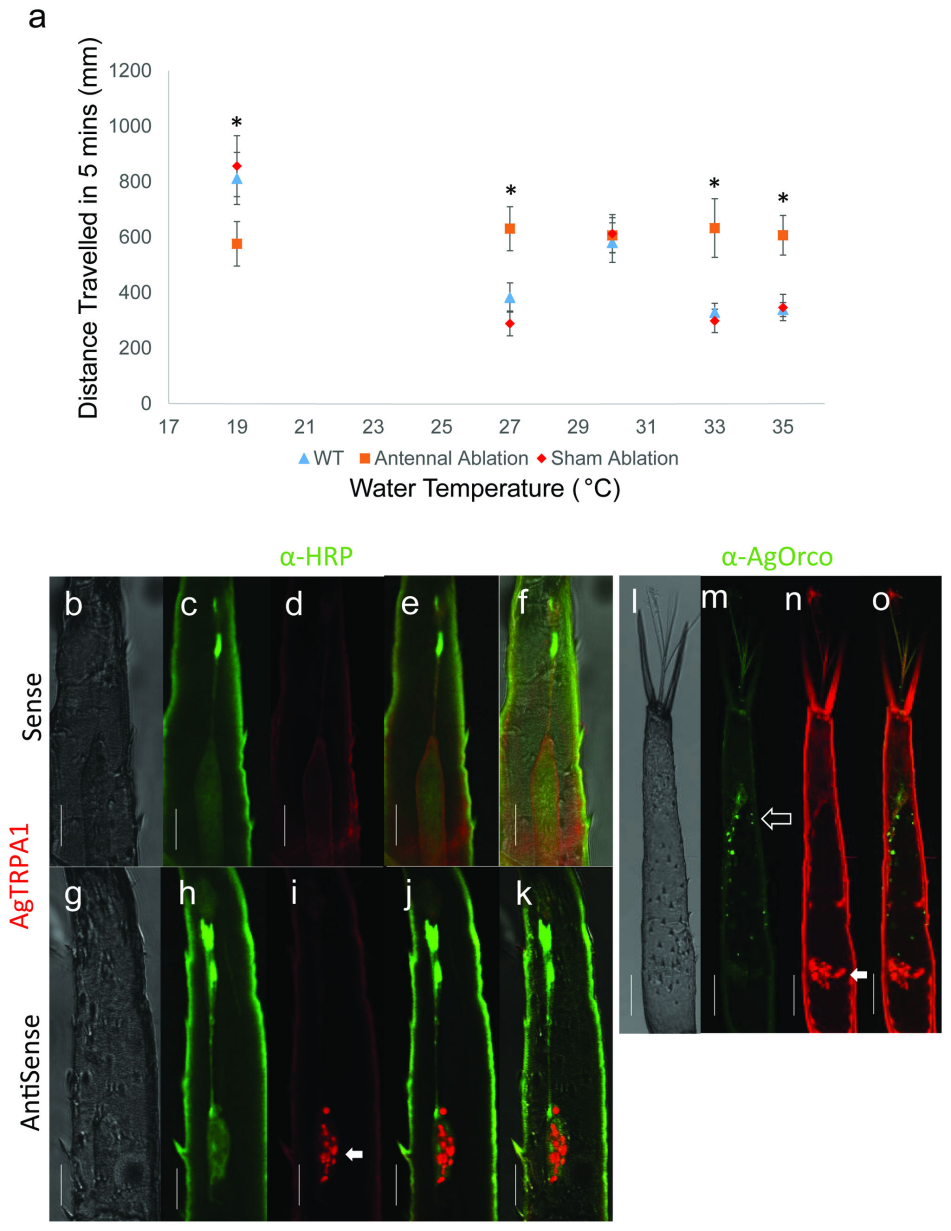
Larval antenna is a peripheral thermosensory organ. **a**) Arithmetic means ± S.E.M of total distance travelled in 300s for individual larva recorded from larvae lacking either antennae or maxillary palp were plotted (n≥12). Asterisks suggest *p*<0.05 using Mann–Whitney *U* test to compare antennal ablation to sham ablation treatment. Kruskal-Wallis one-way analysis of variance was also utilized to compare larval mobility at all 5 temperatures following antennal ablation with *p*>0.05, indicating larvae without antennae were not capable of eliciting differential mobility at varying ambient temperatures comparing to sham treatment. **b**–**k**) Localization of AgTRPA1 mRNA was detected by fluorescent in situ hybridization (FISH). White arrow indicates localization of AgTRPA1 mRNA while green labels neuronal axons and dendrites. **l**–**o**) Red fluorescence indicates AgTRPA1 mRNA while green indicates the localization of AgOrco protein that is expressed in all ORNs. White arrow indicates AgTRPA1-expressing neuronal cell bodies while hollow arrow shows cluster of ORNs (Scale bar, 25µm).

In light of its role in thermosensory processes in adult stage *An. gambiae* [9] and other insects [12], it is reasonable to speculate that AgTRPA1 might also play a role in larval thermosensory pathways. To address this we first carried out RT-PCR studies to confirm the expression of AgTRPA1 in cDNA samples isolated from multiple larval tissues including antennae, head and body where AgTRPA1-specific cDNAs were robustly detected in all tissues (Figure 5). Furthermore, whole-mount fluorescent in situ hybridization (FISH) as well as fluorescent immunohistochemistry-based approaches were used to determine the cellular localization of AgTRPA1 mRNA within larval antennae. These studies (Figure 6g–k) revealed a cluster of 14 AgTRPA1-expressing neuronal cell bodies whose dendrites extend apically. As previous studies in *An. gambiae* larvae discovered a morphologically similar cluster of 12 bi-polar olfactory receptor neuron (ORN) cell bodies [6], we used a polyclonal antisera against the *An. gambiae* odorant receptor co-receptor (AgOrco) which labels all ORNs [6,16] to distinguish putative thermosensory neurons from ORNs. These studies (Figure 6k–n) demonstrate that the AgTRPA1-postive neurons do not overlay or co-localize with the more distal ORN cell cluster on the larval antennae.

In order to further assess the potential role of larval antennae in peripheral thermosensory responses, we carried out behavioral assays following ablation of either the antennae or, as a control, the maxillary palps. In temperature-kinesis studies larvae lacking antennae elicited relatively same level of mobility (total distance: 580-620mm) towards five selected water temperatures which were statistically insignificant from each other (19, 27, 30, 33, 35°C) ranging from cold to hot ambient conditions (Figure 6a). However, in larvae receiving a sham treatment these behavioral responses were statistically indistinguishable from unmanipulated group. Taken together, these data are consistent with the hypothesis that the antenna acts as a peripheral thermosensory appendage that is critical for thermal-induced responses in *An. gambiae* larvae.

**Figure 7:**
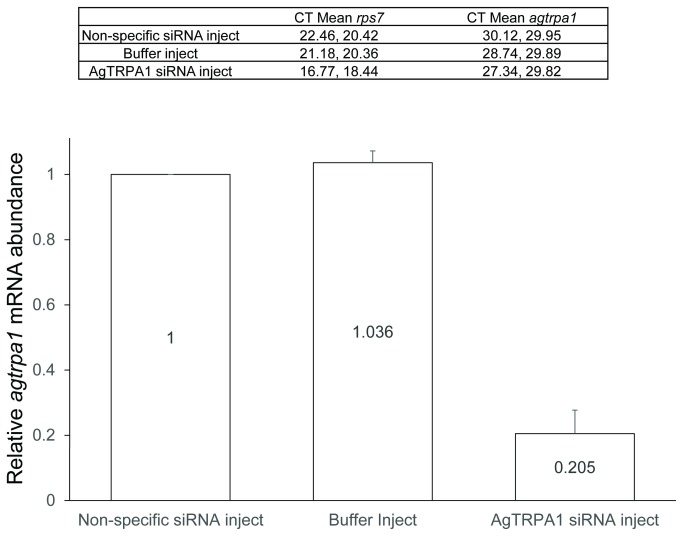
Knockdown of AgTRPA1 mRNA via RNAi. Means of cycle threshold (CT) values for amplification of *agtrpa1* and *rps7* were shown (n=2). Quantitative RT-PCR was performed on cDNA isolated from whole larvae receiving AgTRPA1, non-specific siRNA and buffer injection. Relative mRNA abundance + S.E.M was plotted with data normalized to non-specific siRNA treatment using PFAFFL method.

Due to the absence of available genetic mutants or a viable methodology to generate gene-specific knockouts, we utilized RNAi-mediated gene-silencing to reduce AgTRPA1 mRNA in order to examine the *in vivo* role of AgTRPA1 in larval thermosensation. Small interfering RNA (siRNA) oligonucleotides targeting AgTRPA1 were injected into L3 larvae along with injection of buffer-alone and a non-specific siRNA targeting a gene (AT5G39360) from *Arabidopsis thaliana* that lacks significant homology to *An. gambiae* genome. Knockdown of AgTRPA1 transcript was assessed using quantitative RT-PCR, which showed on average an 80% reduction of mRNA levels (Figure 7) as compared to non-specific siRNA treatment.

**Figure 8:**
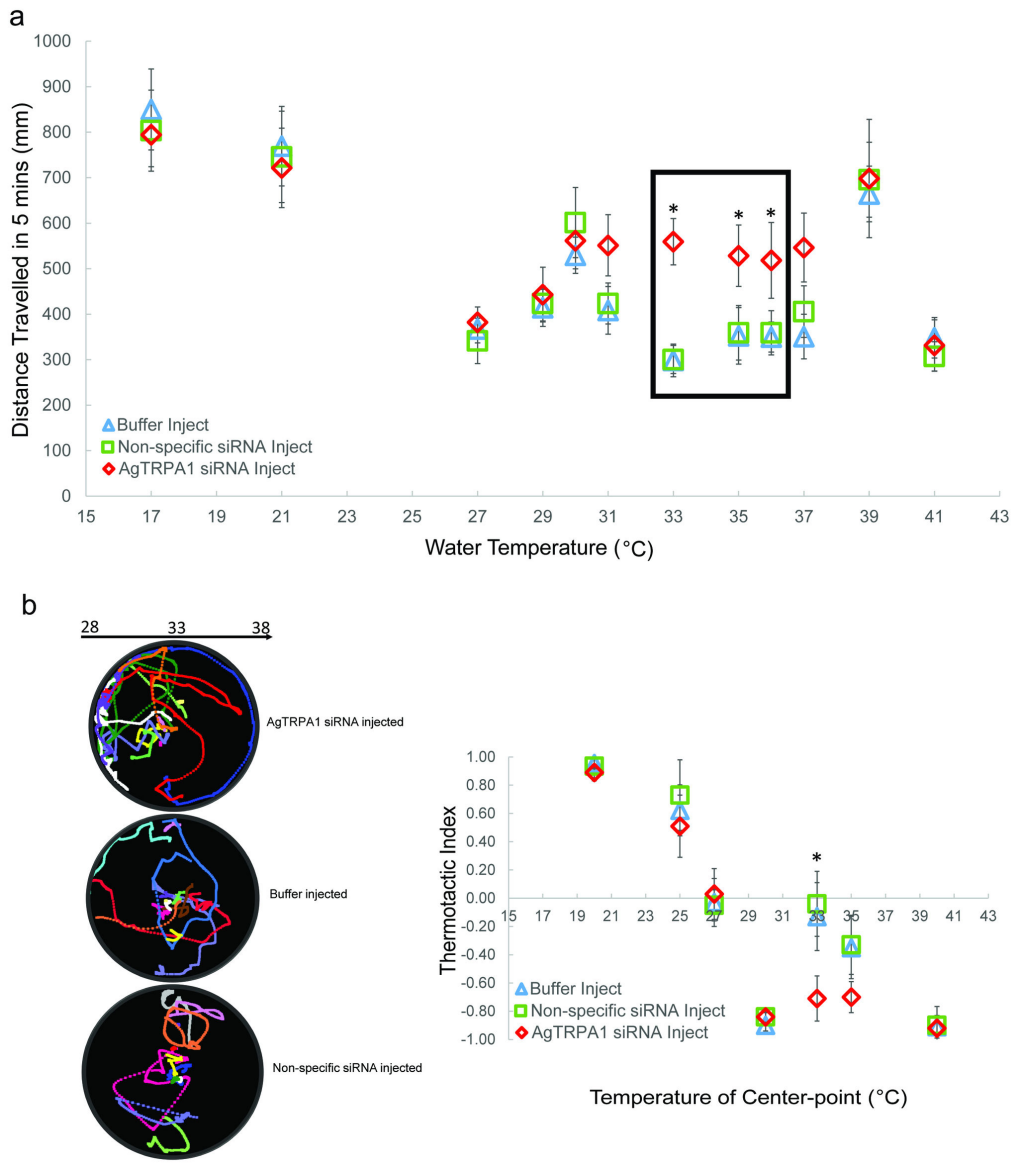
AgTRPA1 mediates larval responses within the upper temperature range. **a**) Arithmetic means ± S.E.M of total distance travelled in 300s for injected larvae reared at 27° C were plotted. Asterisks indicate *p*<0.05 comparing AgTRPA1 and Non-specific siRNA-treatment using Mann–Whitney *U* test. Black rectangle labels the temperature range at which larval mobility was significantly modified following the knockdown of AgTRPA1. **b**) A stack of larval trajectories (n≥10) recorded in 28-38°C gradient for buffer-alone and AgTRPA1, Non-specific siRNA-injected treatments were shown. Thermotactic indices ± S.E.M were plotted for injected larvae. Asterisks indicate *p*<0.05 comparing AgTRAP1 and Non-specific siRNA treatment (Mann–Whitney *U* test).

Behaviorally, *AgTRPA1* knockdown gave rise to a selective effect on larval thermosensory responses that was revealed using both mobility and preference paradigms. In these studies, larval mobility was essentially unaffected relative to controls within the low to mid-temperature ranges while mobility within upper range temperatures (33, 35, 36°C) were significantly increased in AgTRPA1 siRNA-treated larvae (Mann-Whitney *U*, *p*<0.05) (Figure 8a). Similarly, *An. gambiae* larvae receiving AgTRPA1 siRNA showed selective alteration of their thermal preference within the same temperature range where thermotactic indices relative to the non-specific siRNA group decreased at 33 (-0.71±0.16) and 35° C (-0.70±0.11), although the effect achieved at 35° C was statistically insignificant (Figure 8b). These data suggest a role for AgTRPA1 as a selective upper range temperature sensor in *An. gambiae* larvae.

### Larval behavior in the shifted hot range is also AgTRPA1-mediated

**Figure 9:**
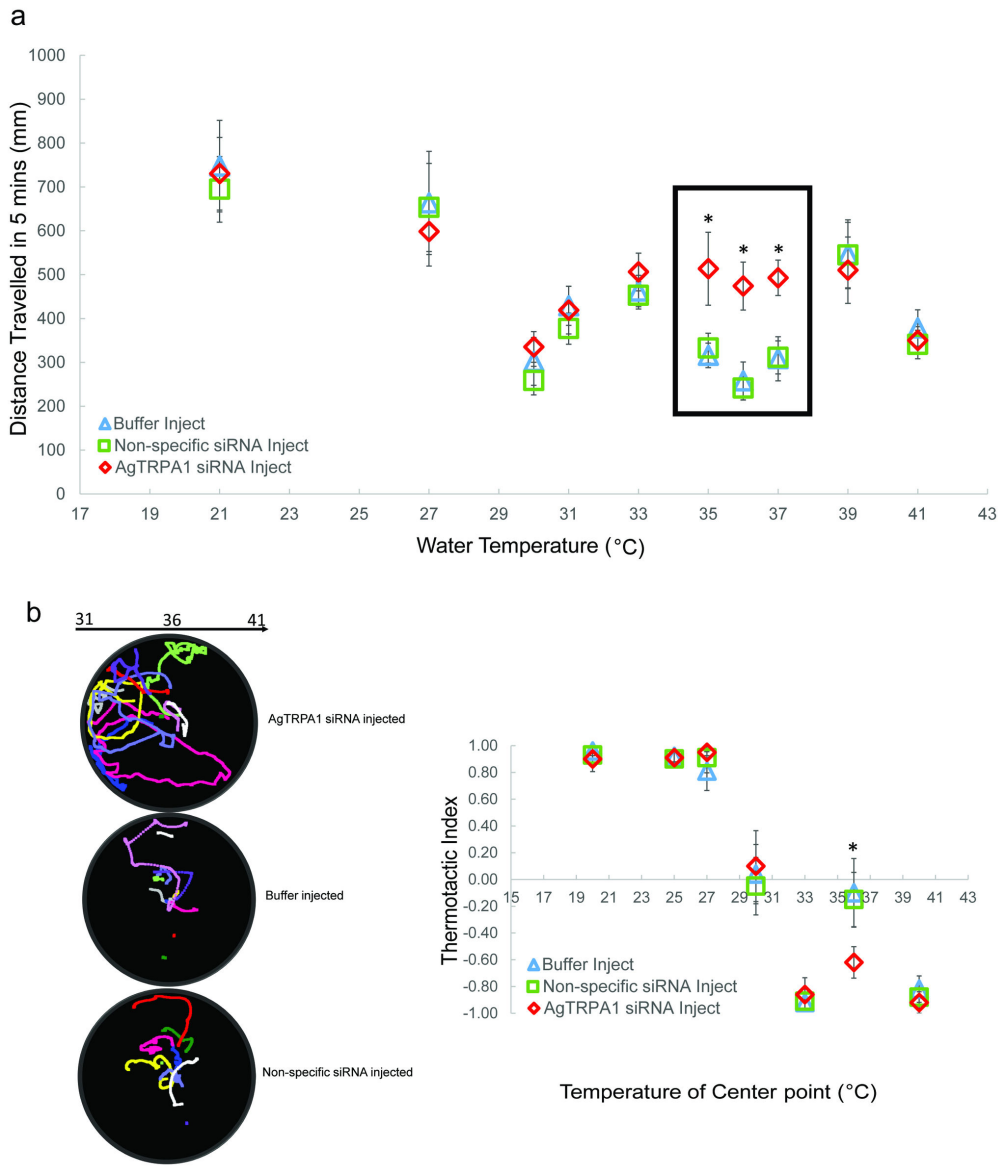
AgTRPA1 mediates larval behavior within the shifted hot range. **a**) Arithmetic means ± S.E.M of total distance travelled in 300s for injected larvae reared at 30° C were plotted. Asterisks indicate *p*<0.05 comparing AgTRAP1 and Non-specific siRNA-injected larvae (Mann–Whitney *U* test). **b**) Stack of larval trajectories (n≥10) recorded in 31-41°C gradient for buffer and AgTRPA1, Non-specific siRNA treatments were shown. Thermotactic indices ± S.E.M were shown for injected larvae reared at 30° C. Asterisks indicate *p*<0.05 comparing AgTRPA1 and Non-specific siRNA-injected larvae (Mann–Whitney *U* test).

To further validate the *in vivo* role of AgTRPA1 in sensing upper range temperatures, we analyzed thermosensory responses in larvae following a 3° C cultivation shift combined with injection of AgTRPA1 siRNA. In kinesis studies, shifted and siRNA-treated larvae displayed normal mobility reductions at their 30° C cultivation point but significantly elevated mobility at 35, 36, 37°C due to the AgTRPA1 knockdown (*p*<0.05, Mann Whitney *U*) (Figure 9a). Similar results were obtained using our thermal gradient assay, where AgTRPA1-dependent hot temperature preference at approximately 6° C above the new 30° C cultivation point was selectively affected by AgTRPA1 silencing whereas larval preferences for the newly shifted cultivation point was still unaffected by AgTRPA1 silencing (Figure 9b).

## Discussion

Together with chemosensory and visual modalities, thermosensory responses of immature *An. gambiae* are necessary for a variety of behaviors pertinent to robust development and survival. Environmental temperature has a major influence on the rate of larval development and, as a result, directly impacts vector populations and malaria transmission [17]. Ambient temperature also influences the growth of algae and bacteria that are the primary nutrients for *An. gambiae* larvae [18]. Although temperature affects the rate of development, the relationship is not straightforward. Indeed, the production of adult mosquitoes is not directly proportional to the rate of larval development such that temperatures resulting in the fastest growth produce fewer and importantly, smaller adults [19]. This reflects the balance between developmental rate and the behaviors that mediate larval survival and feeding in order to obtain adequate dietary reserves which are associated with adult longevity, fecundity and vectorial capacity [20].

Accordingly, throughout larval life-stage, *An. gambiae* effectively navigate across fluctuating water temperatures that might otherwise lead to sub-optimal nutrition, reduced growth and death [19,21]. This capacity is particularly essential for Anopheline larvae in tropical and sub-tropical regions where water temperatures in typical larval habitats with direct sun exposure (i.e. puddles and mud pit) can vary as much as 20° C through day/night cycles [8]. The critical nature of larval thermosensory behaviors underscores the rationale behind studies to characterize the underlying cellular and molecular mechanisms that may, in turn, provide novel opportunities for the development of cost-effective approaches to disrupt these behaviors.

Late-stage *An. gambiae* larvae are capable of responding to diverse temperatures by exhibiting differential kinesis (Figure 1). In our initial survey of thermosensory responses we noted that larval mobility rates are reduced on two occasions, one of which is a discrete point at 27° C followed by a broader interval between 33–36°C that initiates approximately 6° C higher. In light of studies undertaken in other animal systems, these responses may reasonably be associated with behavioral preference while high mobility rates may be correlated with avoidance [13]. Expanding on these observations using a temperature gradient paradigm (Figure 2), we observed that *An. gambiae* larvae are indeed capable of performing thermotactic movements when the surrounding temperature deviates from these favored condition(s).

It is noteworthy that the robust larval preference to 27° C corresponds to their constantly maintained rearing temperature. This is reminiscent of similar observations in *D. melanogaster* where late-stage larvae exhibit maximal growth rate and minimal mortality near 24° C [22] and show behavioral preference towards this temperature when placed on a linear thermal gradient [23]. In order to further investigate the effect of cultivation temperature on larval thermosensory behaviors, we shifted the rearing conditions of a sub-population of newly oviposited *An. gambiae* embryos 3° C higher to 30° C and allowed normal development to proceed to late larval instars. Under these conditions we observed a parallel 3° C shift in larval behavior in both kinesis and thermotaxis bioassays (Figures 3 and 4), suggesting that *An. gambiae* larvae utilize their cultivation conditions to set and adjust their thermal sensors to sense ambient temperatures. Cultivation-induced thermosensory plasticity has been extensively investigated as a behavioral paradigm to elucidate the mechanisms of neural plasticity and learning in the nematode *Caenorhabditis elegans*. In these studies *C. elegans* exhibit thermotaxis towards a new temperature following a short cultivation shift [24]. Similar effects are observed in *D. melanogaster* although alteration of thermal preference required a longer shift of cultivation conditions, typically several days [25]. While the mechanistic basis as to how recalibration of thermal sensors occurs remains unclear, phenotypic plasticity in thermal-driven behavior is crucial for ectotherms where it likely enables them to better adjust to ecological variations [26].

In order to determine the mechanisms for *An. gambiae* larval thermosensory responses, we first carried out antennal ablation on the hypothesis that, as is the case for chemosensation [6], the molecular sensors that detect ambient temperatures to provide input for directing downstream locomotion would likely be associated with this peripheral appendage. This is supported by our ablation studies which demonstrate that *An. gambiae* larvae lacking antennae fail to discriminate between cold and hot ambient conditions across a range of temperatures whilst interestingly maintaining discrete responses to 30° C (Figure 6). It is evident that while a significant proportion of temperature sensors are antennal, additional and as yet cryptic thermosensory signaling pathway(s) exist.

At a molecular level, and in light of its role as a thermosensory receptor on the adult antennae [9], we focused on the role of AgTRPA1 in these processes. In larvae, as in adults, AgTRPA1 transcripts are not restricted to the antennae but also detected in head and body tissues (Figure 5). Within the antennae, AgTRPA1 transcripts localize to a discrete set of proximal neurons that are distinct from the more distal group of AgOrco-expressing ORNs that subtend the sensory cone (Figure 6 [6]). The segregation of olfactory and thermosensory receptor neurons within the antennae is consistent with other Diptera [27].

Studies utilizing siRNA-directed specific gene knockdown reveal that AgTRPA1 is required to maintain thermosensory responses to upper temperature range (Figure 8). When heterologously expressed in *Xenopus* oocytes, AgTRPA1 is detectably activated by temperatures as low as 25° C although robust currents are restricted to stimuli above 30° C [10]. This is consistent with *in vivo* effects where AgTRPA1 knockdown results in larvae that respond normally to cold and warm temperatures but show altered kinesis to hot stimuli between 31 to 37°C, although statistical significance is only achieved at 33, 35 and 36°C (Figure 8a). In addition, AgTRPA1 is essential for larval preferences towards this range of ambient temperatures since the silencing of AgTRPA1 decreases the thermotactic indices at 33 and 35°C. This is similar to the thermosensory threshold of TRPA1 in *D. melanogaster* larvae where dTRPA1 is also activated at moderately elevated temperatures (≥30° C) although in fruit fly, dTRPA1 is required for thermotactic avoidance [28].

The conservation of TRPA1-dependent thermosensory discrimination between *Drosophila* and *Anopheles* larvae in the face of dramatic phenotypic divergence in thermal preference is most likely a consequence of their distinctive terrestrial and aquatic ecology, respectively. In addition, crawling *D. melanogaster* larva biases its forward movements with abrupt reorientation or turns in thermotaxis [29] while swimming *An. gambiae* larvae regulate the distance travelled and latency between repetitive “body twisting” maneuvers [30]. Signaling cascades may have evolved such that thermal stimulation of TRPA1 leads to differential effects on larval motor neurons. Furthermore, the preferred temperature for a given ectotherm is potentially dynamic in and of itself, changing as a function of developmental, environmental or other factors [29].

Larval responses within other thermal ranges, most notably the cultivation point, are not affected by AgTRPA1 silencing and therefore suggest the presence of additional thermal sensors in *An. gambiae*. As is the case in *Drosophila* [31,32], it is likely that in *An. gambiae* multiple molecular sensors, each of which function across a discrete temperature range, act together to transduce thermal information that ultimately lead to downstream behavioral responses.

Taken together, these data demonstrate that thermosensory-mediated behavior in upper-range (“hot”) temperatures in larval stage *An. gambiae* is dependent on the function of AgTRPA1. In addition to characterizing these processes in a biologically important system, these studies support the targeting of AgTRPA1 as a viable approach to interfere with larval development and thereby reduce the vectorial capacity of *An. gambiae*. Natural products such as mustard and horseradish that contain allyl isothiocyanate or cinnamaldehyde, both of which act as potent TRPA1 agonists [33], might be used to develop novel approaches to reduce and/or compromise larval populations of *An. gambiae* and, in doing so, the transmission of human malaria.

## Materials and Methods

### Mosquito rearing and larval sorting

*An. gambiae sensu stricto*, originated from Suakoko, Liberia, was reared as described [34] with modifications for human blood meals described as follows: Five-day old females were allowed to feed on human blood (purchased from Bioeclamation Inc.) for 60 minutes using a Hemotek membrane feeding system (Discovery Workshops, UK) augmented with CO_2_ and human foot odors (derived from a well-worn and unwashed athletic sock), following the guidelines set by Vanderbilt Institutional Animal Care and Use Committee. For behavioral and ablation studies, early 4^th^ or 3^rd^ instar larvae were manually picked out from rearing pan, respectively. Prior to analysis, larvae were rinsed gently with ddH_2_O on a clean metal sieve to remove debris and food residuals and kept in room temperature (24-25°C).

### Thermo-electric control module

In order to generate either homogenous heating or linear temperature gradients in behavioral arena that was composed of glass petri dish of 150mm in diameter filled with 100ml of ddH_2_O, we fabricated a design based on a similar apparatus from [35]. Here, a thin anodized aluminum sheet (12 x 8 x 0.25 inch) was placed on top of two anodized aluminum blocks whose temperatures were adjusted by using both liquid-cooling achieved via water blocks (Custom Thermoelectric) connected to a cycling cold-water bath as well as Peltier devices (Swiftech Inc.) coupled with PID controllers (Oven Industry Inc.). Temperature across the aluminum sheet was set using software (MR001 Ver. Rev B, Oven Industry Inc.). Heating/cooling of each Peltier device was monitored in real-time by dual-mounted thermal probes (Oven Industry Inc.) installed on each end.

### Fluorescent in situ hybridization (FISH) and Fluorescent immunohistochemistry (FIHC) on whole-mount larval antennae

Protocol for FISH studies was adapted and modified from [36]. Briefly, whole larval antennae from 4^th^ instar stage were hand-dissected into 4% PFA in PBS with 0.1% Triton X-100. Samples were then gently transferred into Pyrex glass dish where all subsequent treatments took place. Pre-hybridization and hybridization were performed under 55° C for 6 and 24h, respectively. Fast red staining was used to visualize anti-DIG antibody linked to alkaline phosphatase (AP). Riboprobes were acquired from [9] by amplifying 900bp of *AgTRPA1* coding sequence using PCR primers: Forward: 5′-CTATTCGGCGGCTTCAATAAC-3′ as well as Reverse: 5′-TCATTTGCCAATAGATTTGTTGAAGC-3′. RNA probes were labeled with digoxigenin to generate sense and antisense. Anti-horseradish peroxidase (HRP) antibody conjugated to FITC was utilized to mark neuronal axon and dendrites. Additionally, anti-AgOrco antibody raised from rabbit was used to distinguish between AgTRPA1-expressing neurons and odorant receptor neurons (ORNs). AgOrco labeling was visualized by incubation with Alexa Fluor goat-anti-rabbit 488 (Invitrogen). Whole antennae were mounted in Vectashield (Vector Laboratories) and observed with an LSM510 inverted confocal microscope (Carl Zeiss).

### Automatic larval tracking and analysis

A digital video camera connected to Ethovision XT tracking system (Noldus Inc.) was used to automatically capture and track locomotion of an individual larva in the glass petri dish. For each trial, a single larva was gently introduced at the center of the arena and given 15s to adapt prior to the onset of recording at 10 frames per second (fps). Locomotion was recorded for a total of 300s. For each temperature setting, a minimum of 15 trials (across an equal number of different individuals) was acquired and parameters such as total distance travelled were calculated using Ethovision software. For antennae as well as palp ablation studies, all manipulations were carried out by manual dissection at 3^rd^ instar stages, after which larvae were allowed to recover for 24h prior to behavioral testing. To quantify thermal preferences, we recorded the time interval that each larva spent in either warm or cold half of a gradient that is expressed as thermotactic index (T.I) and calculated as follows:

(t^warm^-t^cold^) / (t^warm^+t^cold^). A negative index value reflects a situation where larvae are more inclined to stay in the cold half of the gradient (negative thermotaxis) whereas a positive value is indicative of the opposite. For statistical analysis, the comparison of two groups was carried out using Mann–Whitney *U* tests while comparison of multiple groups was achieved using Kruskal-Wallis one-way analysis of variance. *p*<0.05 was considered significantly different.

### siRNA injection and quantitative RT-PCR

Larval injections were carried out as previously described [5]. 27.6nL of 20µM/L siRNA that target 6^th^ and 10^th^ exon of AgTRPA1 coding region (UAUUGUUGAGCGGAGUGCCAGUU, UUUUUCUCAUUCGGAUACUCGUU) (Thermo Fisher Inc.) were injected into dorsal side of larval thorax using Nanoliter 2000 systems (World Precision Instruments). Injected larvae were allowed to recover in 27 or 30°C with food provided for 48h. Quantitative RT-PCR was performed to verify the quality of gene knockdown. Ribosomal protein S7 (*rps7*) was chosen as internal control and primers used for these genes were: *rps7*: Forward: 5’- GGTGCACCTGGATAAGAACCA-3’ Reverse:

5’- GTTCTCTGGGAATTCGAACG-3’ (Amplicon size: 112bp) and

agtrpa1

Forward: 5’-TATTCGGCGGCTTCAATAAC-3’ Reverse: 5’-GCGTTTGAAGGATTTCCAGA-3’ (Amplicon size: 115bp). PFAFFL method was used to quantify the relative transcript abundance.
